# Regulation of white and brown adipocyte differentiation by RhoGAP DLC1

**DOI:** 10.1371/journal.pone.0174761

**Published:** 2017-03-30

**Authors:** Choon Kiat Sim, Sun-Yee Kim, Reinhard Brunmeir, Qiongyi Zhang, Hongyu Li, Dharmini Dharmasegaran, Carol Leong, Ying Yan Lim, Weiping Han, Feng Xu

**Affiliations:** 1 Singapore Institute for Clinical Sciences, Agency for Science, Technology and Research (A*STAR), Singapore; 2 Laboratory of Metabolic Medicine, Singapore Bioimaging Consortium, A*STAR, Singapore; 3 Department of Biochemistry, Yong Loo Lin School of Medicine, National University of Singapore, Singapore; University of Hong Kong, HONG KONG

## Abstract

Adipose tissues constitute an important component of metabolism, the dysfunction of which can cause obesity and type II diabetes. Here we show that differentiation of white and brown adipocytes requires Deleted in Liver Cancer 1 (DLC1), a Rho GTPase Activating Protein (RhoGAP) previously studied for its function in liver cancer. We identified *Dlc1* as a super-enhancer associated gene in both white and brown adipocytes through analyzing the genome-wide binding profiles of PPARγ, the master regulator of adipogenesis. We further observed that *Dlc1* expression increases during differentiation, and knockdown of *Dlc1* by siRNA in white adipocytes reduces the formation of lipid droplets and the expression of fat marker genes. Moreover, knockdown of *Dlc1* in brown adipocytes reduces expression of brown fat-specific genes and diminishes mitochondrial respiration. *Dlc1*^*-/-*^ knockout mouse embryonic fibroblasts show a complete inability to differentiate into adipocytes, but this phenotype can be rescued by inhibitors of Rho-associated kinase (ROCK) and filamentous actin (F-actin), suggesting the involvement of Rho pathway in DLC1-regulated adipocyte differentiation. Furthermore, PPARγ binds to the promoter of *Dlc1* gene to regulate its expression during both white and brown adipocyte differentiation. These results identify DLC1 as an activator of white and brown adipocyte differentiation, and provide a molecular link between PPARγ and Rho pathways.

## Introduction

Obesity has become a major healthcare issue and causes metabolic diseases such as type II diabetes and cardiovascular complications [1]. Obesity is associated with an increase in the mass of white adipose tissues (WAT). While WAT primarily stores lipids, there is an additional type of adipose tissue, brown adipose tissue (BAT) that has anti-obesity effects by metabolizing lipids through UCP1-mediated uncoupled respiration [2, 3]. BAT and WAT may have opposing metabolic functions but their differentiation share a common intricate transcriptional program brought by a number of master regulators such as peroxisome proliferator–activated receptor gamma (PPARγ) [4]. During differentiation of both types of adipocytes, there is an upregulation of general adipogenic markers such as *Fabp4* [5, 6] and *Adiponectin* (*AdipoQ*) [7, 8], and additionally in BAT only, there is also increased expression of brown fat genes such as *Ucp1* [9], *Cidea* [10], *Elovl3* [11], and mitochondrial *Cox* genes [12]. Expression of these genes allows uncoupled respiration in BAT to utilize lipids for thermogenesis. This process can be further enhanced by cold exposure to activate the β3-adrenergic pathway *in vivo*, or by norepinephrine or other agonists such as isoproterenol *in vitro* [13]. Long-term cold exposure has also been shown to induce differentiation of brite/beige adipocytes, a brown-like population of adipocytes within the subcutaneous WAT depots [12]. These adipocytes also express *Ucp1* and undergo thermogenesis. Currently the list of regulators of white, brown and beige adipogenic differentiation is still far from complete. And bioinformatics approaches are increasingly utilized to identify novel regulators of adipogenesis [14–17]. Among these approaches, the super-enhancer (SE) association analysis has emerged as a useful tool in the search of novel adipogenic factors. SEs are large clusters of typical enhancers and they are stronger in terms of the ability to activate transcription than typical enhancers [18]. These SEs are usually enriched by cell-type-specific master transcription factor binding and therefore can be defined by the genome-wide binding profiles of these master transcription factors [19]. SEs are also found to be associated with genes that define cell identity and this feature provides the mechanistic basis of the SE association analysis. Through this analysis, we identified RREB1 and PIM1 as new factors promoting brown adipogenesis [14], while KLF11 was identified as an essential regulator of human white fat browning [15].

The Rho family of small guanine nucleotide triphosphatases (GTPase) are components of signaling pathways that control actin cytoskeleton and related biological functions such as cell motility, proliferation, and differentiation [20, 21]. Rho GTPases are regulated in a cycle involving guanine nucleotide exchange factors (RhoGEFs) that activate Rho by converting bound GDP to GTP and the opposing GTPase-activating proteins (RhoGAPs) that inactivate Rho by converting bound GTP to GDP [22]. Active Rho activates Rho-associated kinase (ROCK) and catalyzes the formation of filamentous actin (F-actin) stress fibers from monomeric G-actin. The formation of such a complex actin cytoskeleton allows the differentiation of motile cells such as smooth muscle cells through a transcriptional program brought by Myocardin Related Transcription Factor A (MRTF-A) and serum response factor (SRF) [23]. Conversely, differentiation of non-motile cells such as adipocytes requires low levels of active Rho, ROCK and F-actin [24–27], and these could potentially be caused by activity of an upstream RhoGAP. However so far, only one RhoGAP out of about 80, *p190-B*, has been found to be involved in white adipogenic differentiation [28], and none has been found to regulate brown adipogenic differentiation.

DLC1 is a GAP primarily for RhoA and has been mainly studied in cancer research as a tumor suppressor [29]. Aberrant RhoA signaling during inactivation of *Dlc1* has been shown to cause tumor growth and metastasis [30]. Interestingly, PPARγ has been found to increase *Dlc1* expression to inhibit tumor growth [31], and because PPARγ is a master regulator of adipogenic differentiation, we ask whether PPARγ can also regulate *Dlc1* level in adipocytes and affect differentiation. Furthermore, there is evidence to suggest that DLC1 has a role during development. *Dlc1*-null mice die at embryonic stage of E10.5, with growth defects in placenta, brain, heart and neural tube [32, 33]. At this early developmental stage, adipose tissues have not been formed, and this raises the question: could DLC1 have a role in adipose tissues?

Here we show that DLC1 is a positive regulator of white and brown adipocyte differentiation. *Dlc1* gene is associated with PPARγ defined SEs in both white and brown adipocytes. Knockdown of *Dlc1* by siRNA significantly reduced the accumulation of lipid droplets and the expression of fat markers in both adipocytes. In brown adipocytes, there was also reduced mitochondrial respiration as a result of compromised differentiation when *Dlc1* was knocked down. Additionally, a loss of function of *Dlc1* from *Dlc1*^*-/-*^ mouse embryonic fibroblasts totally abolished adipogenic differentiation. A role of Rho-ROCK-(F-actin) pathway in this process was implied because inhibitors of ROCK and F-actin were able to restore the formation of lipid droplets in *Dlc1*^*-/-*^ MEFs. Finally, ChIP-seq and gene expression analyses showed that *Dlc1* is regulated by PPARγ in both white and brown adipocytes. Together, our results provide evidence for DLC1 as a regulator of white and brown adipocyte differentiation, bridging the effects of PPARγ transcriptional regulation and Rho signaling.

## Results

### *Dlc1* Associates with Super-enhancers in both White and Brown Adipocytes

**Fig 1:**
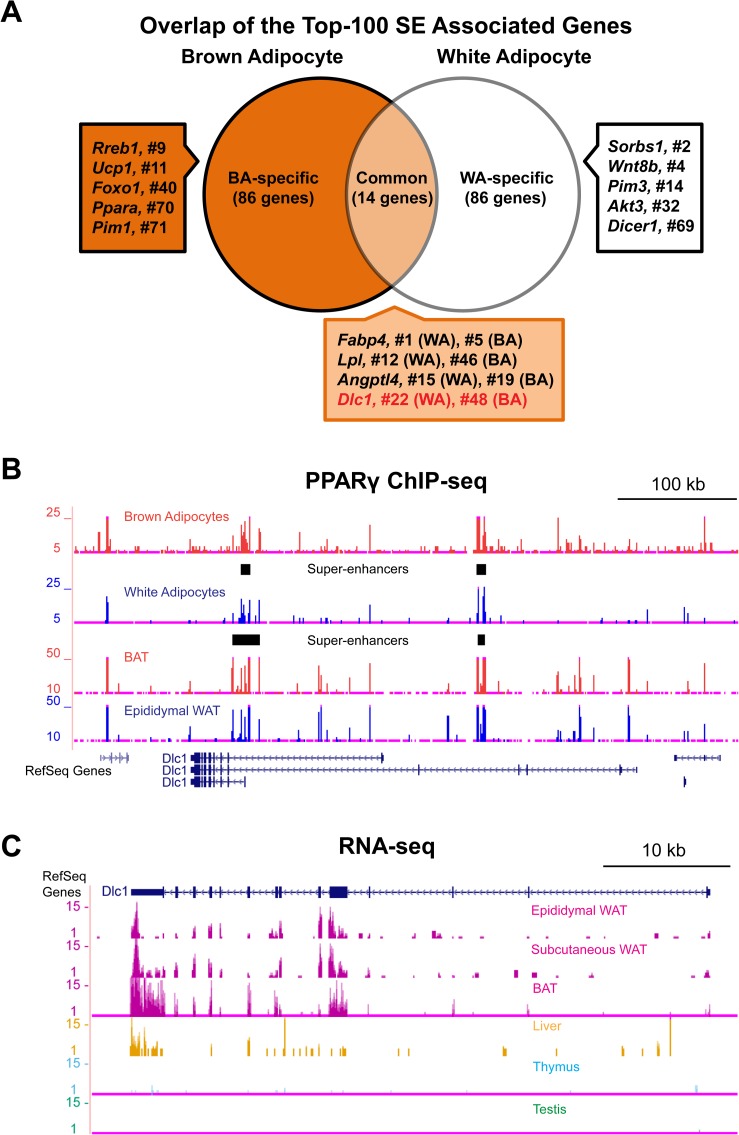
*Dlc1* is Associated with PPARγ Defined Super-enhancers in both White and Brown Adipocytes. **(A)** Venn diagram showing the overlap of the Top-100 super-enhancer associated genes in both white and brown adipocytes. Genome-wide binding profiles of PPARγ were used to define SEs. Representative SE associated genes with their ranks in the corresponding lineages were shown. **(B)** PPARγ binding peaks and their defined SEs at the *Dlc1* gene locus in brown adipocytes, white adipocytes, brown adipose tissue and epididymal white adipose tissue. **(C)** ENCODE RNA-seq data showing the expression of *Dlc1* gene in various mouse tissues.

Recently we profiled PPARγ binding in brown adipocytes [14] differentiated *in vitro* from a mesenchymal stem cell line C3H10T1/2, which was committed to the brown lineage by BMP7 treatment [34]. Using the genome-wide binding profiles of PPARγ in brown adipocytes [14], as well as in 3T3-L1 white adipocytes [16], we defined the SEs in both lineages and identified hundreds of genes associated with these SEs [14]. Among these genes we found most of the established adipogenic markers such as *Pparg*, *Cebpa* and *Fabp4* in both lineages, while we also found the known brown markers including *Ucp1*, *Ppara* and *Cidea* specifically in brown adipocytes [14]. To identify novel adipogenic regulators through this SE association analysis, we selected the Top-100 SE associated genes from both lineages and overlapped these two gene lists (Fig 1A). We found there was an overlap of 14 common genes between these two lists and 86 BA- / WA-specific genes were also identified through this analysis (Fig 1A). As expected, we found most of the brown markers as well as *Rreb1* and *Pim1* in the BA-specific SE associated genes. In the common genes, we found *Fabp4*, *Lpl* and *Angptl4* which were known to have a role in regulating adipogenesis. Interestingly, we also found *Dlc1*, a gene which was not studied in the context of fat cell differentiation, to be associated with SEs in both lineages (Fig 1A and 1B). Moreover, even higher levels of PPARγ binding were observed in both brown adipose tissue and epididymal white adipose tissue, suggesting that *Dlc1* is also regulated by PPARγ defined SEs *in vivo* (Fig 1B). We further examined *Dlc1* expression in various tissues using RNA-seq data from ENCODE and we found that *Dlc1* expression is present in epididymal WAT (eWAT, a visceral fat depot) and subcutaneous WAT and also particularly high in BAT relative to liver, thymus or testis (Fig 1C) [35]. Together, these data strongly suggest that DLC1 has a role in regulating both white and brown adipogenesis.

### DLC1 is required for white adipogenesis

**Fig 2:**
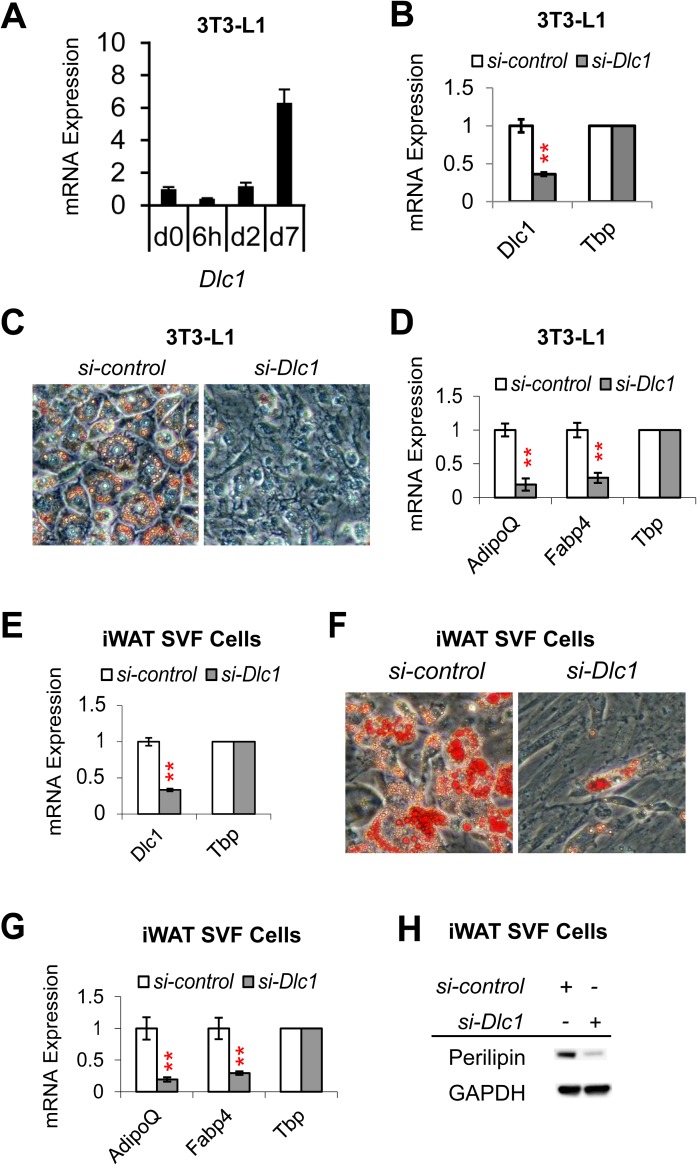
DLC1 is Required for White Adipogenic Differentiation. **(A)** mRNA expression of *Dlc1* gene during differentiation of 3T3-L1 white adipocytes. **(B)** qRT-PCR result showing the knockdown of *Dlc1* gene expression in 3T3-L1 white adipocytes. *Tbp* was used as normalizing control for gene expression. **(C)** Oil-Red-O staining showing reduced lipid droplet formation in *Dlc1* knockdown cells. **(D)** mRNA expression of adipogenic marker genes *Adiponectin* (*AdipoQ*) and *Fabp4* in *si-Dlc1* white adipocytes. **(E)** qRT-PCR result showing the knockdown of *Dlc1* gene expression in primary iWAT SVF cells derived white adipocytes. **(F)** Oil-Red-O staining showing reduced lipid droplet formation in primary iWAT SVF cell derived white adipocytes upon *Dlc1* knockdown. **(G)** mRNA expression of adipogenic marker genes *Adiponectin* and *Fabp4* in *si-Dlc1* white adipocytes derived from primary iWAT SVF cells. **(H)** Western blotting analysis of mature adipocyte marker perilipin in primary iWAT SVF cell derived white adipocytes upon *Dlc1* knockdown. Data are presented as mean ± s.e.m. *n* = 3–4 biological replicates. Two-tailed Student’s *t*-test was used: ** *P* < 0.01.

To examine *Dlc1* expression during white adipogenesis *in vitro*, we used a classic WA cell line 3T3-L1. We found that *Dlc1* expression increased by 6-fold during differentiation of 3T3-L1 cells, suggesting that the gene may be required for white adipogenesis (Fig 2A). To test if *Dlc1* is indeed required for white adipogenesis, we knocked down the expression of *Dlc1* using siRNA (i.e. *si-Dlc1*) in 3T3-L1 cells (Fig 2B) and examined the impact on adipogenesis efficiency by both Oil-Red-O staining and qRT-PCR of adipogenic markers. As shown in Fig 2C, *Dlc1* knockdown led to a significant reduction of lipid accumulation in 3T3-L1 cells. Moreover, classic adipogenic marker genes such as *Adiponectin* and *Fabp4* were also reduced by *Dlc1* knockdown (Fig 2D). To test the function of *Dlc1* in primary white adipocytes, we isolated the Stromal Vascular Fraction (SVF) cells from inguinal white fat and knocked down *Dlc1* before adipogenic induction (Fig 2E). As determined by Oil-Red-O staining (Fig 2F), there was a large decrease in lipid accumulation hence adipogenesis efficiency in iWAT SVF cells by *si-Dlc1*. And this was accompanied by reduced expression of adipogenic genes *Adiponectin* and *Fabp4* in *si-Dlc1* cells (Fig 2G). Furthermore, the reduced efficiency of differentiation was correlated with a decrease in the amount of perilipin [36], a mature adipocyte marker that associates with the surface of lipid droplets (Fig 2H). In summary, these results indicate that *Dlc1* is necessary for the differentiation of white adipocytes.

### DLC1 is required for brown adipogenesis

**Fig 3:**
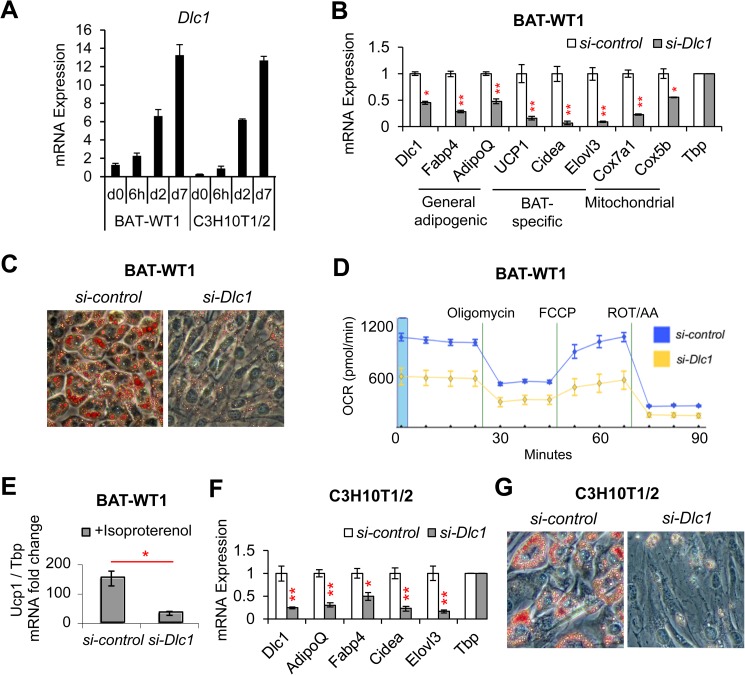
DLC1 Regulates Brown Adipogenesis and Brown Cell Function. **(A)** mRNA expression of *Dlc1* gene during differentiation of BAT-WT1 brown adipocytes and C3H10T1/2 derived brown adipocytes. **(B)** mRNA expression of *Dlc1*, general adipogenic, BAT-specific and mitochondrial genes in BAT-WT1 brown adipocytes upon *Dlc1* knockdown. **(C)** Oil-Red-O staining showing reduced lipid droplet formation in *Dlc1* knockdown BAT-WT1 brown adipocytes. **(D)** Oxygen consumption rates (OCR) in BAT-WT1 brown adipocytes with or without *Dlc1* knockdown. Vertical green lines indicate the time points of Oligomycin, FCCP and Rotenone/Antimycin A (ROT/AA) injection. *n* = 3. **(E)** qRT-PCR measurements of the fold change of *Ucp1* expression after isoproterenol treatment compared with basal levels without treatment. *Dlc1* knockdown significantly impaired the upregulation of *Ucp1* expression after isoproterenol treatment. **(F)** mRNA expression of *Dlc1*, general adipogenic and BAT-specific genes in *Dlc1* knockdown brown adipocytes derived from C3H10T1/2 cells. **(G)** Oil-Red-O staining showing reduced lipid droplet formation in *Dlc1* knockdown brown adipocytes derived from C3H10T1/2 cells. Data are presented as mean ± s.e.m. *n* = 3–6 biological replicates. Two-tailed Student’s *t*-test was used: * *P* < 0.05, ** *P* < 0.01.

We next asked whether DLC1 is also required for brown adipogenesis. To check *Dlc1* expression during brown adipogenesis *in vitro*, we used two BA differentiation models: C3H10T1/2 cells pretreated with BMP7 [34] and BAT-WT1 cells (an immortalized BA preadipocyte cell line derived from mouse BAT) [37]. We found that *Dlc1* expression increased significantly during differentiation of both cell lines, suggesting that *Dlc1* is important for brown adipogenic differentiation (Fig 3A). To test the function of *Dlc1* in brown adipogenesis, we knocked down this gene in both cell lines (Fig 3B and 3F) and observed that *Dlc1* knockdown reduced differentiation of both BAT-WT1 cells (Fig 3C) and C3H10T1/2 cells (Fig 3G). For BAT-WT1 cells, there were not only reductions in expression of general adipogenic genes such as *Fabp4* and *Adiponectin* but also BAT-specific genes such as *Ucp1*, *Cidea*, *Elovl3*, and mitochondrial genes such as *Cox7a1* and *Cox5b* (Fig 3B). We also asked whether the reduced differentiation and BAT/mitochondrial gene expression could affect mitochondrial respiration. In this regards we measured the oxygen consumption rate (OCR) of the cells and observed that basal, uncoupled and maximal respiration—the latter two stimulated by oligomycin (ATP synthase inhibitor) and carbonyl cyanide-*p-*trifluoromethoxyphenylhydrazone (FCCP; mitochondrial uncoupler), respectively—were reduced by *si-Dlc1* (Fig 3D). As brown cells are known to be involved in thermogenesis, we added isoproterenol, an activator of the β3-adrenergic pathway, to stimulate BA thermogenesis. We found that *Ucp1*, a classic marker of thermogenesis, was significantly reduced in *si-Dlc1* cells compared with *si-control* cells (Fig 3E), suggesting that *si-Dlc1* also reduced thermogenesis. The lower mitochondrial respiration and thermogenesis were likely secondary to the reduced differentiation of brown adipocytes. For C3H10T1/2 cells, we also observed reductions in expression of general adipogenic genes as well as BAT-specific genes upon *Dlc1* knockdown (Fig 3F). Finally, to exclude the possibility that the phenotype observed in *Dlc1* knockdown cells is derived from off-target effect of the *Dlc1* siRNA used in Fig 2 and Fig 3, we tested an additional siRNA of *Dlc1* and observed a similar reduction in adipogenesis and decreases in adipogenic and brown marker gene expression (S1 Fig). Taken together, these results suggest that *Dlc1* is required for brown adipogenesis.

### *Dlc1* heterozygous mice show no phenotype in adipose tissues and whole-body metabolism

To test the adipogenic function of DLC1 *in vivo*, we acquired the *Dlc1* heterozygous (*Dlc1/+*) mice from the Popescu lab [32]. As the homozygous *Dlc1* mutant is embryonically lethal, we examined the *Dlc1/+* mice for possible metabolic phenotypes. Our results suggest that *Dlc1/+* mice showed no difference in total body mass, lean mass or fat mass (S2A Fig), or the masses of BAT, inguinal WAT (iWAT) or eWAT (S2B Fig). Microscopically there was no obvious difference in the Hematoxylin and Eosin (H&E) staining of BAT or iWAT between wild-type and *Dlc1/+* (S2C Fig) nor was there significant molecular difference in terms of UCP1 level in BAT or iWAT between the two genotypes (S2E Fig). To test whether there are differences in energy expenditure at the animal physiology level, mice were placed individually in metabolic chambers where food intake, ambulatory movement, oxygen consumption, carbon dioxide production were measured, and the latter two were used to calculate respiratory exchange ratio (RER) and energy expenditure using indirect calorimetry. We noticed that there was no significant difference in these parameters (S3 Fig). To study whether DLC1 could affect the adipogenic differentiation of brite/beige adipocytes, we subjected mice to a temperature of 4°C for a week to induce the inguinal fat depot to generate beige adipocytes that express UCP1. In wild-type mice, the low temperature reduced the lipid content in BAT and also produced beige adipocytes with multilocular lipid droplets in the iWAT compared with wild-type mice kept at room temperature (S2C and S2D Fig). However even at low temperature (4°C), both adipose tissues from *Dlc1/+* mice were indistinguishable from wild-type using H&E staining (S2D Fig) and UCP1 protein level (S2F Fig). Particularly, this data from iWAT suggested that *Dlc1/+* mice have no defect in beige adipogenic differentiation. Together, there appeared to be no obvious phenotype in white, brown and beige adipose tissues as well as whole-body metabolism in the *Dlc1* heterozygous mouse.

### Adipogenic role of Dlc1 involves the rho pathway

**Fig 4:**
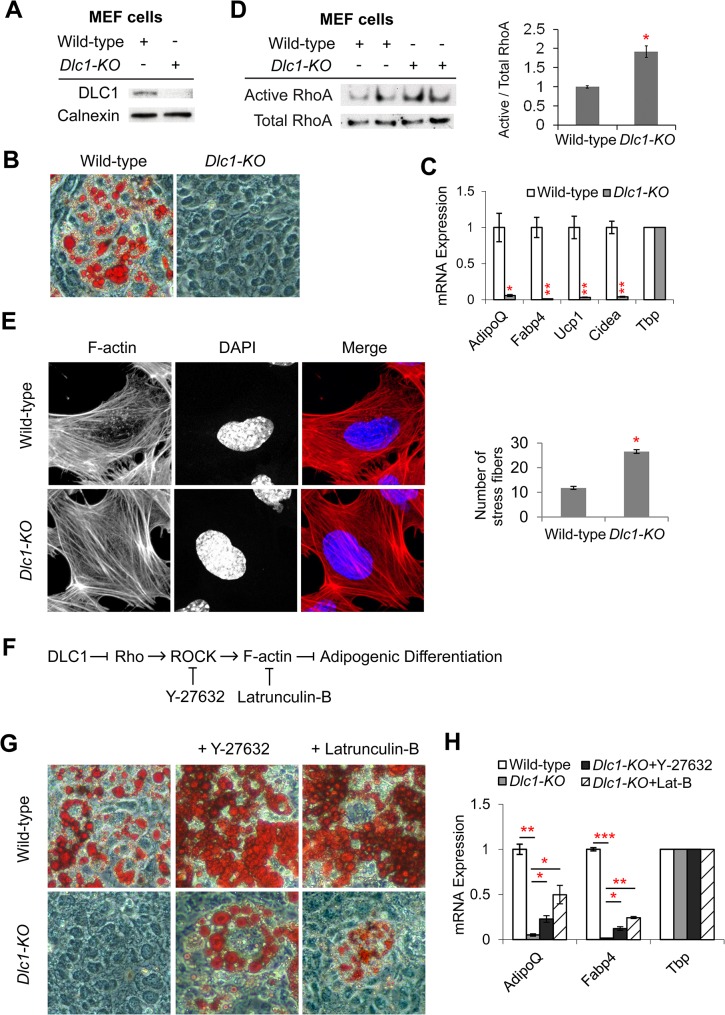
Adipogenic Role of DLC1 Involves the Rho Pathway. **(A)** Western blots showing the absence of DLC1 protein in *Dlc1-KO* MEFs. Calnexin was included as a loading control. **(B)** Oil-Red-O staining of differentiated wild-type and *Dlc1-KO* MEFs. **(C)** Relative mRNA expression of adipogenic and BAT-specific genes in differentiated MEFs. **(D)** Western blots showing active RhoA levels in *Dlc1-KO* MEFs. Total RhoA was shown as a loading control. Band intensity was quantified using ImageJ and the ratio between Active / Total RhoA was presented as bar graph in the right panel. **(E)** Phalloidin staining of F-actin in MEFs (left panels) and quantification of F-actin stress fibers (right panel). *n* = 15. **(F)** Proposed model of DLC1-Rho pathway and the site of action for ROCK inhibitor Y-27632 and F-actin inhibitor Latrunculin-B. **(G)** Oil-Red-O staining to show the rescue of lipid droplet formation by the ROCK and F-actin inhibitors in *Dlc1-KO* MEFs. **(H)** qRT-PCR showing the restoration of adipogenic gene expression. Data are presented as mean ± s.e.m. *n* = 2–3 biological replicates. Two-tailed Student’s *t*-test was used: * *P* < 0.05, ** *P* < 0.01, *** *P* < 0.001.

A possible explanation for the lack of metabolic phenotype in *Dlc1* heterozygous mice is that the presence of one copy of the *Dlc1* gene might already be sufficient for normal adipose tissue development and function. To study the impact of DLC1 ablation on adipogenic differentiation in cells with both copies of the *Dlc1* gene removed, we turned to mouse embryonic fibroblast (MEF). *Dlc1*^*-/-*^ (or *Dlc1-KO*) embryo dies at E10.5 stage [32], so we isolated embryos at E9.5. Using a SV40 large T antigen protocol, we immortalized the MEF cells and differentiated these cells into brown-like adipocytes. First we confirmed the absence of DLC1 protein in *Dlc1-KO* MEFs by western blotting (Fig 4A). We also noticed that while wild-type MEFs differentiated into lipid-laden adipocytes, the *Dlc1-KO* MEFs failed to produce any mature adipocyte (Fig 4B). Gene expression analysis showed that the *Dlc1-KO* cells had substantially lower levels of adipogenic markers *Adiponectin* and *Fabp4* relative to wild-type cells (Fig 4C). In addition, brown marker genes *Ucp1* and *Cidea* were also significantly down-regulated by *Dlc1* knockout (Fig 4C).

To investigate the mechanism by which *Dlc1-KO* led to defects in adipogenic differentiation, we considered that DLC1 protein has three domains: an N-terminal sterile alpha motif (SAM) domain, a RhoGAP domain, and a C-terminal steroidogenic acute regulatory protein (StAR)- related lipid-transfer (START) domain. The SAM domain allows protein-protein interaction, while the START domain has a role in lipid transport or lipid metabolism [29]. In this manuscript, we focused on studying the RhoGAP function of DLC1. As DLC1 causes the conversion of active Rho-GTP to inactive Rho-GDP, we expect that *Dlc1-KO* MEFs have deficiency in this conversion and thus have higher levels of active Rho. We measured the amount of active RhoA and found that indeed there was higher level of active RhoA in the *Dlc1-KO* MEFs (Fig 4D). This result agrees with a previous literature which showed that active Rho is associated with an undifferentiated cell state [25]. As a high level of active RhoA is also associated with an increased formation of F-actin stress fibers especially those found in the center of the cells [38], we also checked and found a higher number of central stress fibers in *Dlc1-KO* MEFs (Fig 4E). To ask whether the adipogenic regulation by DLC1 acts through the Rho pathway, we subjected differentiating *Dlc1-KO* MEFs to treatment with either Y-27632 (an inhibitor of Rho*-*associated kinase/ROCK), or Latrunculin-B (an inhibitor of F-actin assembly). ROCK and F-actin formation are effectors of the Rho pathway and normally inhibits adipogenic differentiation (Fig 4F) [25–27, 39]. We reasoned that ROCK and F-actin are downstream of DLC1 in the Rho pathway, and their inhibition will rescue the effects of *Dlc1-KO* and increase adipogenic differentiation. Indeed, we found that both Y-27632 and Latrunculin-B treatments provided partial rescue, as seen in an increase in both lipid droplets (Fig 4G) and adipogenic gene expression (Fig 4H). These results corroborate the findings obtained using *Dlc1* siRNA and suggest that DLC1 is an important regulator of adipogenic differentiation through the Rho pathway.

### *Dlc1* is a PPARγ-target gene

**Fig 5:**
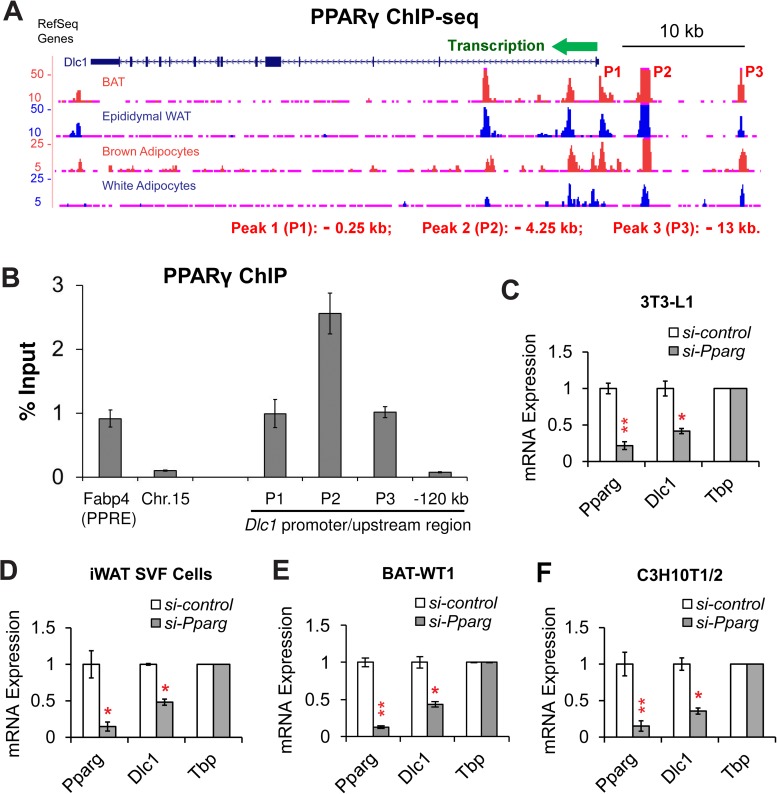
*Dlc1* is a Direct Target of PPARγ. **(A)** PPARγ ChIP-seq data revealed direct binding of this master regulator of adipogenesis at the promoter of *Dlc1* gene in mouse BAT, eWAT, 3T3-L1 white adipocytes and C3H10T1/2 derived brown adipocytes. The positions (Relative to the transcription start site of *Dlc1* gene) of PPARγ binding peaks (P1-P3) are indicated under the graph. **(B)** PPARγ ChIP confirmed its binding to Peak 1–3 at the promoter/ upstream region of *Dlc1* gene in C3H10T1/2 cells (Brown adipocytes). *Fabp4* gene promoter and a chromosome 15 (Chr.15) region were used here as positive/negative controls for PPARγ ChIP. **(C-F)** qRT-PCR results showing the knockdown of *Pparg* and the reduced expression of *Dlc1* gene in **(C)** 3T3-L1 white adipocytes, **(D)** primary iWAT SVF cell derived white adipocytes, **(E)** BAT-WT1 brown adipocytes and **(F)** C3H10T1/2 derived brown adipocytes. Data are presented as mean ± s.e.m. *n* = 3–6 biological replicates. Two-tailed Student’s *t*-test was used: * *P* < 0.05, ** *P* < 0.01.

We next investigated the upstream transcriptional regulator of *Dlc1* during adipogenesis. Previous super-enhancer analysis suggested that *Dlc1* associates with PPARγ defined super-enhancers in both white and brown fat cells, indicating an essential role for this master regulator of adipogenesis in controlling *Dlc1* expression. Therefore, we examined PPARγ binding at the promoter of *Dlc1* gene. Our analysis of published ChIP-seq datasets revealed that PPARγ bound to the promoter of *Dlc1* gene in mouse BAT, eWAT [17], 3T3-L1 white adipocytes [40] and C3H10T1/2 derived brown adipocytes [14] at multiple positions (Fig 5A). Three major PPARγ binding peaks (P1-P3) were identified at position—0.25 kb, - 4.25 kb and—13 kb upstream of the transcription start site (TSS) of *Dlc1* gene. Moreover, our PPARγ binding ChIP in C3H10T1/2 cells (Brown adipocytes) confirmed that PPARγ indeed binds to P1-P3 at the promoter / upstream region of *Dlc1* gene but not a region—120 kb upstream of the *Dlc1* TSS (Fig 5B). *Fabp4* gene promoter which contains a PPAR response element (PPRE) was used as a positive control [41] for PPARγ ChIP, while a chromosome 15 (Chr.15) region devoid of any known genes was included as a negative control [42]. PPARγ is known to regulate the transcription of an array of adipogenic genes during differentiation. To test whether *Dlc1* is a target gene of PPARγ, we conducted a knockdown of *Pparg* expression using siRNA in 3T3-L1 white adipocytes, primary iWAT SVF cell derived white adipocytes, BAT-WT1 brown adipocytes and C3H10T1/2 derived brown adipocytes (Fig 5C–5F). We found that *Pparg* knockdown caused a reduction of *Dlc1* expression in all fat cells examined (Fig 5C–5F), suggesting that PPARγ is upstream of *Dlc1* and directly regulates *Dlc1* expression during both brown and white adipogenic differentiation.

## Discussion

We have shown that DLC1 is essential for the differentiation of both white and brown adipocytes. The phenotype was observed using *Dlc1* siRNA in two types of white adipocytes and two types of brown adipocytes, and was even more noticeable in *Dlc1-KO* MEFs with a complete absence of lipid droplets. These lipid droplets were nonetheless restored by the treatment of inhibitors of Rho-associated kinase/ROCK and F-actin, implicating the Rho pathway in DLC1 regulation. We also showed that adipogenic regulator PPARγ was recruited to the gene promoter of *Dlc1* to regulate its expression in both white and brown adipocytes. Together, the results support the importance of DLC1 in white and brown adipogenic differentiation.

Recently, the Rho/ROCK pathway has been a subject of intense study, as inhibition of the components of Rho/ROCK pathway was shown to increase adipogenic differentiation of WAT, BAT and beige fat [25–27, 39]. Moreover, downstream of Rho and ROCK is a transcriptional program led by SRF and MRTF-A that target about a thousand genes involved in cytoskeleton, cell growth and transcription [43], regulating cell lineage between adipocyte and smooth muscle [27, 44, 45]. As DLC1 is a RhoGAP, the involvement of the Rho pathway in the regulation of DLC1 in adipogenic differentiation is in line with the function of DLC1 as a RhoGAP, but we have nevertheless shown that DLC1 is involved in both white and brown fat differentiation and is likely to be part of the core machinery controlling the regulation of Rho. Further work can be pursued to test whether and how DLC1 affects downstream MRTF-A/SRF and the switch of cell lineages.

There are about 80 RhoGAPs in the genome and it is a curious question how many of these proteins could play a role in adipogenic differentiation. While RhoGAP p190-B is known to regulate adipogenic differentiation [28], DLC2 (or STARD13), a closely related protein to DLC1, was found to be not involved [46]. Thus it is likely that there is some form of selection of specific RhoGAP proteins for adipogenic differentiation. Even if these proteins are supposed to act on RhoA or other Rho-related GTPases, there could be contextual differences in their functions such as temporal, subcellular localization, tissue-specific cofactors or post-translational protein modifications that restrict their access to Rho or modify their activity. A systematic characterization of each RhoGAP in adipogenic differentiation could help to resolve some of these questions.

The rescue of lipid droplets in *Dlc1-KO* MEFs by the treatment of ROCK inhibitor and F-actin inhibitor suggests that the Rho pathway is involved in the regulation of adipogenic differentiation by DLC1, but it might not be the only mechanism. First, this rescue is only partial as seen from the expression analyses. Second, the upregulation of active RhoA in *Dlc1-KO* is small and might not be able to account for the total abolishment of adipogenic differentiation of *Dlc1-KO* MEFs. To resolve this paradox, we note that DLC1 does have non-Rho related functions, including the binding to structural proteins such as caveolin lipid rafts [47], adapter/scaffolding protein 14-3-3 [48, 49], or elongation factor EF1A1 [50], These non-Rho functions of DLC1 might also affect adipogenic differentiation and could be explored in greater details.

PPARγ is a master regulator of general adipogenic differentiation as seen for its roles in white, brown, and beige adipocytes [51–53]. While different adipocytes vary in post-translational modifications of PPARγ or tissue-specific recruitment of cofactors such as PRDM16, the common function of PPARγ in all types of adipocytes is to activate the expression of many genes involved in lipid and glucose homeostasis, adipokine production and insulin signaling [54]. Our results suggest that *Dlc1* is also a PPARγ -target gene and is directly involved in the process of adipogenic differentiation. Further work can be pursued to test whether DLC1 also affects lipid metabolism, adipokine production or insulin signaling. In particular, DLC1 has a START domain that was found to be important in lipid transport or lipid metabolism in other proteins [29, 55]. Potentially the START domain could bind lipids and regulate subcellular localization of the protein. Other START proteins bind a large range of lipid ligands such as cholesterol, phospholipids, sphingolipids, oxysterols and fatty acids [55], and it is interesting to find which ligand binds to DLC1.

In conclusion, our findings have provided additional insights into the regulation of adipocyte differentiation. The discovery of DLC1 as both a white and a brown adipogenic regulator shows its importance in the fundamental development of the two tissues. This is a helpful step towards improving our treatment options to ameliorate obesity and bring metabolic balance.

## Materials and methods

### Mouse studies

This study was carried out in strict accordance with the recommendations in the Guide for the Care and Use of Laboratory Animals of the National Institutes of Health. The protocol was approved by the Institutional Animal Care and Use Committee of the Agency for Science, Technology and Research (A-STAR) of Singapore (IACUC#: 140956). *Dlc1* heterozygous mice were in C57BL6/J:129Sv genetic background and were housed on a 12-hour light-dark cycle with access to water and normal chow diet (Harlan 2018 Teklab Global 18% Protein Rodent Diet). For cold exposure studies, mice were placed at 4°C in single cages for a week.

### Indirect calorimetry

Metabolic rates of mice were measured by the Oxymax / Comprehensive Lab Animal Monitoring System (Columbus Instrument, Ohio). Mice were kept in individual chambers at 24°C under a 12-hour light/dark cycle. Mice were acclimatized for a day and then for two days, oxygen consumption (VO2), carbon dioxide production (VCO2), food intake and physical movement were measured. Respiratory exchange ratio (RER) was calculated as the ratio VCO2/VO2 and estimates which fuel is metabolized to produce energy (0.7 = pure fat; 1 = pure carbohydrate). Energy expenditure was calculated as (3.815+ 1.232 x RER) x VO2 and normalized to lean mass.

### Body fat mass and lean mass

Body fat mass and lean mass were measured by using EchoMRI-100 (Echo Medical Systems).

### Histology

Tissues were fixed in 10% neutral-buffered formalin, embedded in paraffin, and stained with hematoxylin and eosin. Slides were viewed using a Leica DM6000B microscope.

### MEF isolation and immortalization

Timed mating was performed using *Dlc1* heterozygous females with heterozygous males. Embryos were removed from pregnant females after 9.5 days, individually genotyped, trypsinized to single cells and cultured. Wild-type and *Dlc1-KO* MEFs were immortalized using SV40 large T antigen (Addgene plasmid # 21826). The plasmid is transfected into primary MEF using lipofectamine 2000 (Thermo Fisher) and cells were passaged using the “3T3” method for ~10 times until immortalized cells appear. At least two wild-type and two knockout lines were tested in experiments.

### Primary SVF cell isolation from inguinal WAT

Inguinal stroma-vascular fraction containing preadipocytes was isolated from whole inguinal fat depot. Briefly, adipose tissue was dispersed into single cells using 1 mg/ml collagenase II (Sigma) + 20 mg/ml BSA in PBS for 1 hour, incubated with red blood cell lysis buffer (Biolegend) for 10 min, and filtered through 100 μm strainer.

### Cell culture

(A) BAT-WT1 preadipocytes, a gift from Kai Ge lab and originally from Yu-Hua Tseng/ Ronald Kahn lab, were cultured and differentiated as previously described [56]. Cells were maintained in 10% Fetal Bovine Serum (HyClone) in Dulbecco’s modified Earle’s medium (DMEM) at 37°C in 5% CO_2_. To differentiate into mature adipocytes, cells were cultured in 1 nM triiodothyronine (T3) and 20 nM insulin for three days, switched to medium containing 1 nM T3, 20 nM insulin, 0.5 mM isobutylmethylxanthine (IBMX), 5 μM dexamethasone (DEX), 125 μM indomethacin for two days and then at every two days thereafter, replaced with medium containing 1 nM T3 and 20 nM insulin. Cells were harvested at day 7. To stimulate thermogenesis, cells were treated with 10 μM isoproterenol for 3 hours.

(B) C3H10T1/2 cells were cultured and differentiated as previously described [34]. Cells were cultured in 8.3 nM BMP7 for 3 days and then differentiated using 0.5 mM IBMX, 5 μM DEX, 1 nM T3, 20 nM insulin, 1 μM rosiglitazone for 2 days and subsequently every two days, the medium was changed to 1 nM T3, 20 nM insulin, and 1 μM rosiglitazone. Cells were harvested at day 9.

(C) 3T3-L1 preadipocytes were cultured and differentiated as described [57]. Cells were cultured to confluency for 3 days and induced with differentiation medium containing 1 μM DEX, 0.5 mM IBMX, and 10 μg/ml insulin for two days. Subsequently every two days, cells were incubated with fresh medium containing 10 μg/ml insulin. Cells were harvested at day 9.

(D) iWAT SVF cells were cultured in DMEM/F12 (Thermo Fisher) with 20% FBS and used for seeding by the fourth day of harvest. For differentiation, cells were treated with 5 μM DEX, 85 nM insulin, 0.5 mM IMBX, 50 nM rosiglitazone, 1 nM triiodothyronine (T3) for three days, and then transferred to medium containing 85 nM insulin and 1 nM T3 for another day before the cells are harvested.

(E) MEFs were cultured in 10% Fetal Clone III in DMEM. For differentiation to adipocytes, MEFs were treated with 8 nM BMP7 for 3 days, and then switched to medium containing 1 μM rosiglitazone, 1 nM T3, 20 nM insulin, 125 μM indomethacin, 5 μM dexamethasone and 250 μM isobutylmethylxanthine (IBMX) for 2 days. Medium was then changed to 1 μM rosiglitazone, 1 nM T3, 20 nM insulin for 2 days, and refreshed for another 2 more days. This cycle of (6 drugs for 2 days and 3 drugs for 4 days) was done for a total of three cycles until day 18, and then the 3 drugs were added for ten more days until day 28 when cells were harvested. For the rescue experiments to study the involvement of Rho pathway, 25 μM ROCK inhibitor Y-27632 or 300 nM Latrunculin-B were added whenever the 3 drugs were added.

### siRNA knockdown

For *Dlc1* and *Pparg* knockdown studies, transfection of siRNA (Exiqon) was done using lipofectamine 2000 (Thermo Fisher) a day before differentiation and cells were harvested at day 3 or 4 post-differentiation. Control siRNA is scrambled siRNA of the same chemical backbone.

### qRT-PCR

Total RNA was isolated using Trizol and purified with RNeasy mini kit (Qiagen). cDNA synthesis was done using M-MLV reverse transcriptase (Thermo Fisher). Quantitative PCR was performed using Power SYBR Green PCR master mix (Applied Biosystems) or TaqMan Universal Master Mix II (Applied Biosystems) with the 7900HT Fast Real-Time PCR System (Applied Biosystems). *Tbp* was used as normalizing control for gene expression. Three biological replicates were done unless otherwise stated.

### Western blot

Mouse tissues were minced and sonicated in RIPA buffer containing protease inhibitor, and after maximal centrifugation, the clear portion of the supernatant was loaded onto the gel. Primary antibodies used were anti-Perilipin (1: 1000, Cell Signaling), anti-GAPDH (1: 20,000, Cell Signaling), anti-DLC1 (#612020, BD Bioscience) and anti-Calnexin (SC-11397, Santa Cruz).

### Oil-Red-O staining

Oil-Red-O staining was performed as described previously [56]. Briefly, cells were fixed in 4% formaldehyde in PBS for 20 minutes and incubated in 3 mg/ml Oil-Red-O solution in 60% isopropanol for an hour.

### Immunostaining

Immunostaining was performed essentially as described [58]. Cells were seeded on coverslips and cultured as per normal. During harvest, cells were fixed in 4% paraformaldehyde/PBS for 20 min and permeabilized for 1 hour in 0.6% Triton X-100/PBS. For F-actin staining, cells were incubated for 20 minutes in phalloidin rhodamine (1:40, Molecular Probes) in 10% goat serum/ 0.1% Triton X-100/PBS. Cells were imaged using Nikon Eclipse A1 confocal microscopy in z-stack over 3 μm at 0.2 μm interval and maximally projected to a plane. Stress fibers were counted using the method described [59]. Briefly, ImageJ was used to draw a line along the short axis of the cell and over the nucleus. Each peak in the plot profile of the line was counted as a stress fiber.

### Oxygen consumption rate

Oxygen consumption rates (OCR) were measured using a Seahorse extracellular flux analyzer XF24e (Seahorse Bioscience). Prior to measurement, cells were equilibrated in assay medium containing 25 mM glucose and 1 mM sodium pyruvate for an hour. To measure OCR due to mitochondrial respiration, cells were treated sequentially with 4 μM oligomycin, 0.2 μM carbonyl cyanide-*p-*trifluoromethoxyphenylhydrazone (FCCP), and 1 μM rotenone/antimycin A.

### Active Rho assay

The level of active Rho in wild-type or *Dlc1-KO* MEFs was measured using the Active Rho Pull-down and Detection kit (Thermo Scientific). Briefly, a GST-fusion of the Rhotekin binding domain (RBD) was used to pull down active Rho (Rho-GTP) and the eluted sample was probed using Western Blot with an anti-RhoA antibody (1:200, Santa Cruz) to identify the amount of active RhoA.

### ChIP

Chromatin immunoprecipitation (ChIP) assay was performed as described recently [14]. In brief, cells were crosslinked with 1% formaldehyde for 10 min at 37°C, then crude nuclei were purified and sonicated using a Bioruptor UCD-300 (Diagenode) to obtain chromatin fragments approximately 500 bp in length. PPARγ antibody (#2443, Cell Signaling) was added at 3 μl per ChIP and incubated overnight at 4°C. ChIP and input DNA were quantified by real-time quantitative PCR analysis using SYBR green and a 7900HT fast real-time PCR system (Applied Biosystems).

### Genomic data

RNA-seq data were obtained from ENCODE with these GEO accessions: epididymal/genital fat (GSM900190), subcutaneous fat (GSM900191), or UCSC accessions: BAT (wgEncodeEM002635), liver (wgEncodeEM001714), and testis (wgEncodeEM002389). PPARγ ChIP-seq data were obtained from [17] with GEO accession GSE43763, and from [16] with GEO accession GSE20752.

### Statistical analysis

All statistics were performed using two-tailed Student’s *t*-test using *p*-value < 0.05 for significance. Data were presented as mean values ± s.e.m.

## Supporting information

S1 Fig A second siRNA targeting *Dlc1* reduces adipogenesis and the expression of adipogenic and brown marker genes. (A) Oil-Red-O staining showing reduced lipid droplet formation in *Dlc1* knockdown BAT-WT1 brown adipocytes using a second *Dlc1* siRNA. (B) mRNA expression of *Dlc1*, general adipogenic, BAT-specific genes in BAT-WT1 brown adipocytes upon Dlc1 knockdown using a second *Dlc1* siRNA. Data are presented as mean ± s.e.m. n = 3 biological replicates. Two-tailed Student’s t-test was used: * P < 0.05, ** P < 0.01.
(TIF)

S2 Fig *Dlc1* heterozygous mice show no phenotype in adipose tissues. (A) Fat mass, lean mass, and total body mass of 8-week-old male *Dlc1/+* and wild-type mice. *n* = 10. (B) Mass of BAT, inguinal WAT (iWAT), and epididymal WAT (eWAT). (C-D) H&E staining of BAT and iWAT of mice kept at 25°C (C) or 1 week at 4°C (D). (E-F) Western blot showing UCP1 level in BAT and iWAT of mice kept at 25°C (E) and 1 week at 4°C (F).
(TIF)

S3 Fig *Dlc1* heterozygous mice show no phenotype in whole-body metabolism. (A-D) Energy expenditure (A), respiratory exchange ratio (RER) (B), physical activity (C), and food intake (D) in wild-type and *Dlc1/+* mice. *n* = 6.
(TIF)

S1 Table List of the top-100 super-enhancer associated genes in brown and white adipocytes. (XLSX)

## References

[1] Singla P, Bardoloi A, Parkash AA. Metabolic effects of obesity: A review. World J Diabetes. 2010;1(3):76–88. Epub 2011/05/04. 10.4239/wjd.v1.i3.76 21537431 PMC3083889

[2] Tang QQ, Lane MD. Adipogenesis: from stem cell to adipocyte. Annu Rev Biochem. 2012;81:715–36. Epub 2012/04/03. 10.1146/annurev-biochem-052110-115718 22463691

[3] Kajimura S, Saito M. A new era in brown adipose tissue biology: molecular control of brown fat development and energy homeostasis. Annu Rev Physiol. 2014;76:225–49. Epub 2013/11/06. 10.1146/annurev-physiol-021113-170252 24188710 PMC4090362

[4] Ahmadian M, Suh JM, Hah N, Liddle C, Atkins AR, Downes M, et al PPARgamma signaling and metabolism: the good, the bad and the future. Nat Med. 2013;19(5):557–66. Epub 2013/05/09. 10.1038/nm.3159 23652116 PMC3870016

[5] Spiegelman BM, Frank M, Green H. Molecular cloning of mRNA from 3T3 adipocytes. Regulation of mRNA content for glycerophosphate dehydrogenase and other differentiation-dependent proteins during adipocyte development. J Biol Chem. 1983;258(16):10083–9. 6411703

[6] Hunt CR, Ro JH, Dobson DE, Min HY, Spiegelman BM. Adipocyte P2 gene: developmental expression and homology of 5'-flanking sequences among fat cell-specific genes. Proc Natl Acad Sci U S A. 1986;83(11):3786–90. PubMed Central PMCID: PMCPMC323608. 3520554 10.1073/pnas.83.11.3786 PMC323608

[7] Scherer PE, Williams S, Fogliano M, Baldini G, Lodish HF. A novel serum protein similar to C1q, produced exclusively in adipocytes. J Biol Chem. 1995;270(45):26746–9. 7592907 10.1074/jbc.270.45.26746

[8] Hu E, Liang P, Spiegelman BM. AdipoQ is a novel adipose-specific gene dysregulated in obesity. J Biol Chem. 1996;271(18):10697–703. 8631877 10.1074/jbc.271.18.10697

[9] Enerback S, Jacobsson A, Simpson EM, Guerra C, Yamashita H, Harper ME, et al Mice lacking mitochondrial uncoupling protein are cold-sensitive but not obese. Nature. 1997;387(6628):90–4. 10.1038/387090a0 9139827

[10] Zhou Z, Yon Toh S, Chen Z, Guo K, Ng CP, Ponniah S, et al Cidea-deficient mice have lean phenotype and are resistant to obesity. Nature genetics. 2003;35(1):49–56. 10.1038/ng1225 12910269

[11] Westerberg R, Mansson JE, Golozoubova V, Shabalina IG, Backlund EC, Tvrdik P, et al ELOVL3 is an important component for early onset of lipid recruitment in brown adipose tissue. J Biol Chem. 2006;281(8):4958–68. 10.1074/jbc.M511588200 16326704

[12] Harms M, Seale P. Brown and beige fat: development, function and therapeutic potential. Nat Med. 2013;19(10):1252–63. 10.1038/nm.3361 24100998

[13] Wu J, Cohen P, Spiegelman BM. Adaptive thermogenesis in adipocytes: is beige the new brown? Genes Dev. 2013;27(3):234–50. Epub 2013/02/08. PubMed Central PMCID: PMC3576510. 10.1101/gad.211649.112 23388824 PMC3576510

[14] Brunmeir R, Wu J, Peng X, Kim SY, Julien SG, Zhang Q, et al Comparative Transcriptomic and Epigenomic Analyses Reveal New Regulators of Murine Brown Adipogenesis. PLoS genetics. 2016;12(12):e1006474 PubMed Central PMCID: PMCPMC5140063. 10.1371/journal.pgen.1006474 27923061 PMC5140063

[15] Loft A, Forss I, Siersbaek MS, Schmidt SF, Larsen AS, Madsen JG, et al Browning of human adipocytes requires KLF11 and reprogramming of PPARgamma superenhancers. Genes Dev. 2015;29(1):7–22. PubMed Central PMCID: PMCPMC4281566. 10.1101/gad.250829.114 25504365 PMC4281566

[16] Mikkelsen TS, Xu Z, Zhang X, Wang L, Gimble JM, Lander ES, et al Comparative epigenomic analysis of murine and human adipogenesis. Cell. 2011;143(1):156–69. Epub 2010/10/05. 10.1016/j.cell.2010.09.006 PMC2950833 20887899

[17] Rajakumari S, Wu J, Ishibashi J, Lim HW, Giang AH, Won KJ, et al EBF2 determines and maintains brown adipocyte identity. Cell Metab. 2013;17(4):562–74. PubMed Central PMCID: PMC3622114. 10.1016/j.cmet.2013.01.015 23499423 PMC3622114

[18] Whyte WA, Orlando DA, Hnisz D, Abraham BJ, Lin CY, Kagey MH, et al Master transcription factors and mediator establish super-enhancers at key cell identity genes. Cell. 2013;153(2):307–19. PubMed Central PMCID: PMC3653129. 10.1016/j.cell.2013.03.035 23582322 PMC3653129

[19] Hnisz D, Abraham BJ, Lee TI, Lau A, Saint-Andre V, Sigova AA, et al Super-enhancers in the control of cell identity and disease. Cell. 2013;155(4):934–47. PubMed Central PMCID: PMC3841062. 10.1016/j.cell.2013.09.053 24119843 PMC3841062

[20] Sit ST, Manser E. Rho GTPases and their role in organizing the actin cytoskeleton. J Cell Sci. 2011;124(Pt 5):679–83. Epub 2011/02/16. 10.1242/jcs.064964 21321325

[21] Jaffe AB, Hall A. Rho GTPases: biochemistry and biology. Annu Rev Cell Dev Biol. 2005;21:247–69. Epub 2005/10/11. 10.1146/annurev.cellbio.21.020604.150721 16212495

[22] Cherfils J, Zeghouf M. Regulation of small GTPases by GEFs, GAPs, and GDIs. Physiol Rev. 2013;93(1):269–309. Epub 2013/01/11. 10.1152/physrev.00003.2012 23303910

[23] Olson EN, Nordheim A. Linking actin dynamics and gene transcription to drive cellular motile functions. Nat Rev Mol Cell Biol. 2010;11(5):353–65. Epub 2010/04/24. 10.1038/nrm2890 20414257 PMC3073350

[24] McBeath R, Pirone DM, Nelson CM, Bhadriraju K, Chen CS. Cell shape, cytoskeletal tension, and RhoA regulate stem cell lineage commitment. Dev Cell. 2004;6(4):483–95. Epub 2004/04/08. 15068789 10.1016/s1534-5807(04)00075-9

[25] Nobusue H, Onishi N, Shimizu T, Sugihara E, Oki Y, Sumikawa Y, et al Regulation of MKL1 via actin cytoskeleton dynamics drives adipocyte differentiation. Nat Commun. 2014;5:3368 Epub 2014/02/27. 10.1038/ncomms4368 24569594

[26] Noguchi M, Hosoda K, Fujikura J, Fujimoto M, Iwakura H, Tomita T, et al Genetic and pharmacological inhibition of Rho-associated kinase II enhances adipogenesis. J Biol Chem. 2007;282(40):29574–83. Epub 2007/08/08. 10.1074/jbc.M705972200 17681946

[27] McDonald ME, Li C, Bian H, Smith BD, Layne MD, Farmer SR. Myocardin-related transcription factor A regulates conversion of progenitors to beige adipocytes. Cell. 2015;160(1–2):105–18. Epub 2015/01/13. 10.1016/j.cell.2014.12.005 25579684 PMC4384505

[28] Sordella R, Jiang W, Chen GC, Curto M, Settleman J. Modulation of Rho GTPase signaling regulates a switch between adipogenesis and myogenesis. Cell. 2003;113(2):147–58. Epub 2003/04/23. 12705864 10.1016/s0092-8674(03)00271-x

[29] Durkin ME, Yuan BZ, Zhou X, Zimonjic DB, Lowy DR, Thorgeirsson SS, et al DLC-1:a Rho GTPase-activating protein and tumour suppressor. J Cell Mol Med. 2007;11(5):1185–207. Epub 2007/11/06. 10.1111/j.1582-4934.2007.00098.x 17979893 PMC4401278

[30] Lukasik D, Wilczek E, Wasiutynski A, Gornicka B. Deleted in liver cancer protein family in human malignancies (Review). Oncol Lett. 2011;2(5):763–8. Epub 2012/08/07. 10.3892/ol.2011.345 22866123 PMC3408103

[31] Bren-Mattison Y, Van Putten V, Chan D, Winn R, Geraci MW, Nemenoff RA. Peroxisome proliferator-activated receptor-gamma (PPAR(gamma)) inhibits tumorigenesis by reversing the undifferentiated phenotype of metastatic non-small-cell lung cancer cells (NSCLC). Oncogene. 2005;24(8):1412–22. Epub 2004/12/21. 10.1038/sj.onc.1208333 15608671

[32] Durkin ME, Avner MR, Huh CG, Yuan BZ, Thorgeirsson SS, Popescu NC. DLC-1, a Rho GTPase-activating protein with tumor suppressor function, is essential for embryonic development. FEBS Lett. 2005;579(5):1191–6. Epub 2005/02/16. 10.1016/j.febslet.2004.12.090 15710412

[33] Sabbir MG, Wigle N, Loewen S, Gu Y, Buse C, Hicks GG, et al Identification and characterization of Dlc1 isoforms in the mouse and study of the biological function of a single gene trapped isoform. BMC Biol. 2010;8:17 Epub 2010/03/05. 10.1186/1741-7007-8-17 20199662 PMC2839985

[34] Tseng YH, Kokkotou E, Schulz TJ, Huang TL, Winnay JN, Taniguchi CM, et al New role of bone morphogenetic protein 7 in brown adipogenesis and energy expenditure. Nature. 2008;454(7207):1000–4. PubMed Central PMCID: PMC2745972. 10.1038/nature07221 18719589 PMC2745972

[35] Consortium EP. An integrated encyclopedia of DNA elements in the human genome. Nature. 2012;489(7414):57–74. Epub 2012/09/08. 10.1038/nature11247 22955616 PMC3439153

[36] Greenberg AS, Egan JJ, Wek SA, Garty NB, Blanchette-Mackie EJ, Londos C. Perilipin, a major hormonally regulated adipocyte-specific phosphoprotein associated with the periphery of lipid storage droplets. J Biol Chem. 1991;266(17):11341–6. 2040638

[37] Klein J, Fasshauer M, Ito M, Lowell BB, Benito M, Kahn CR. beta(3)-adrenergic stimulation differentially inhibits insulin signaling and decreases insulin-induced glucose uptake in brown adipocytes. J Biol Chem. 1999;274(49):34795–802. Epub 1999/11/27. 10574950 10.1074/jbc.274.49.34795

[38] Katoh K, Kano Y, Ookawara S. Rho-kinase dependent organization of stress fibers and focal adhesions in cultured fibroblasts. Genes Cells. 2007;12(5):623–38. Epub 2007/05/31. 10.1111/j.1365-2443.2007.01073.x 17535253

[39] Haas B, Mayer P, Jennissen K, Scholz D, Berriel Diaz M, Bloch W, et al Protein kinase G controls brown fat cell differentiation and mitochondrial biogenesis. Sci Signal. 2009;2(99):ra78 Epub 2009/12/03. 10.1126/scisignal.2000511 19952371

[40] Mikkelsen TS, Xu Z, Zhang X, Wang L, Gimble JM, Lander ES, et al Comparative epigenomic analysis of murine and human adipogenesis. Cell. 2010;143(1):156–69. Epub 2010/10/05. 10.1016/j.cell.2010.09.006 20887899 PMC2950833

[41] van Beekum O, Brenkman AB, Grontved L, Hamers N, van den Broek NJ, Berger R, et al The adipogenic acetyltransferase Tip60 targets activation function 1 of peroxisome proliferator-activated receptor gamma. Endocrinology. 2008;149(4):1840–9. Epub 2007/12/22. 10.1210/en.2007-0977 18096664

[42] Zhang Q, Ramlee MK, Brunmeir R, Villanueva CJ, Halperin D, Xu F. Dynamic and distinct histone modifications modulate the expression of key adipogenesis regulatory genes. Cell Cycle. 2012;11(23):4310–22. PubMed Central PMCID: PMC3552913. 10.4161/cc.22224 23085542 PMC3552913

[43] Esnault C, Stewart A, Gualdrini F, East P, Horswell S, Matthews N, et al Rho-actin signaling to the MRTF coactivators dominates the immediate transcriptional response to serum in fibroblasts. Genes Dev. 2014;28(9):943–58. Epub 2014/04/16. 10.1101/gad.239327.114 24732378 PMC4018493

[44] Liao XH, Wang N, Liu QX, Qin T, Cao B, Cao DS, et al Myocardin-related transcription factor-A induces cardiomyocyte hypertrophy. IUBMB Life. 2011;63(1):54–61. Epub 2011/02/01. 10.1002/iub.415 21280178

[45] Long JZ, Svensson KJ, Tsai L, Zeng X, Roh HC, Kong X, et al A smooth muscle-like origin for beige adipocytes. Cell Metab. 2014;19(5):810–20. Epub 2014/04/09. 10.1016/j.cmet.2014.03.025 24709624 PMC4052772

[46] Yau TO, Leung TH, Lam S, Cheung OF, Tung EK, Khong PL, et al Deleted in liver cancer 2 (DLC2) was dispensable for development and its deficiency did not aggravate hepatocarcinogenesis. PLoS One. 2009;4(8):e6566 Epub 2009/08/12. 10.1371/journal.pone.0006566 19668331 PMC2718616

[47] Yamaga M, Sekimata M, Fujii M, Kawai K, Kamata H, Hirata H, et al A PLCdelta1-binding protein, p122/RhoGAP, is localized in caveolin-enriched membrane domains and regulates caveolin internalization. Genes Cells. 2004;9(1):25–37. Epub 2004/01/16. 14723705 10.1111/j.1356-9597.2004.00698.x

[48] Scholz RP, Regner J, Theil A, Erlmann P, Holeiter G, Jahne R, et al DLC1 interacts with 14-3-3 proteins to inhibit RhoGAP activity and block nucleocytoplasmic shuttling. J Cell Sci. 2009;122(Pt 1):92–102. Epub 2008/12/11. 10.1242/jcs.036251 19066281

[49] Lim GE, Albrecht T, Piske M, Sarai K, Lee JT, Ramshaw HS, et al 14-3-3zeta coordinates adipogenesis of visceral fat. Nat Commun. 2015;6:7671 Epub 2015/07/30. 10.1038/ncomms8671 26220403 PMC4532800

[50] Zhong D, Zhang J, Yang S, Soh UJ, Buschdorf JP, Zhou YT, et al The SAM domain of the RhoGAP DLC1 binds EF1A1 to regulate cell migration. J Cell Sci. 2009;122(Pt 3):414–24. Epub 2009/01/23. 10.1242/jcs.027482 19158340

[51] Koppen A, Kalkhoven E. Brown vs white adipocytes: the PPARgamma coregulator story. FEBS Lett. 2010;584(15):3250–9. Epub 2010/07/06. 10.1016/j.febslet.2010.06.035 20600006

[52] Qiang L, Wang L, Kon N, Zhao W, Lee S, Zhang Y, et al Brown Remodeling of White Adipose Tissue by SirT1-Dependent Deacetylation of Ppargamma. Cell. 2012;150(3):620–32. Epub 2012/08/07. PubMed Central PMCID: PMC3413172. 10.1016/j.cell.2012.06.027 22863012 PMC3413172

[53] Ohno H, Shinoda K, Spiegelman BM, Kajimura S. PPARgamma agonists induce a white-to-brown fat conversion through stabilization of PRDM16 protein. Cell Metab. 2013;15(3):395–404. Epub 2012/03/13. 10.1016/j.cmet.2012.01.019 PMC3410936 22405074

[54] Janani C, Ranjitha Kumari BD. PPAR gamma gene—a review. Diabetes Metab Syndr. 2015;9(1):46–50. Epub 2014/12/03. 10.1016/j.dsx.2014.09.015 25450819

[55] Clark BJ. The mammalian START domain protein family in lipid transport in health and disease. J Endocrinol. 2012;212(3):257–75. Epub 2011/10/04. 10.1530/JOE-11-0313 21965545

[56] Gao M, Sim CK, Leung CW, Hu Q, Feng G, Xu F, et al A fluorescent light-up probe with AIE characteristics for specific mitochondrial imaging to identify differentiating brown adipose cells. Chem Commun (Camb). 2014;50(61):8312–5. Epub 2014/06/19. 24940580 10.1039/c4cc00452c

[57] Kim SY, Sim CK, Tang H, Han W, Zhang K, Xu F. Acetylome study in mouse adipocytes identifies targets of SIRT1 deacetylation in chromatin organization and RNA processing. Arch Biochem Biophys. 2016;598:1–10. 10.1016/j.abb.2016.03.025 27021582

[58] Sim CK, Perry S, Tharadra SK, Lipsick JS, Ray A. Epigenetic regulation of olfactory receptor gene expression by the Myb-MuvB/dREAM complex. Genes Dev. 2012;26(22):2483–98. Epub 2012/10/30. 10.1101/gad.201665.112 23105004 PMC3505819

[59] Strachan LR, Condic ML. Neural crest motility and integrin regulation are distinct in cranial and trunk populations. Dev Biol. 2003;259(2):288–302. Epub 2003/07/23. 12871702 10.1016/s0012-1606(03)00187-8

