# Supplementary figures and images for: Regulation of white and brown adipocyte differentiation by RhoGAP DLC1
Source: PLoS One. 2017 Mar 30;12(3):e0174761. doi: 10.1371/journal.pone.0174761 (PMC5373604; doi:10.1371/journal.pone.0174761)

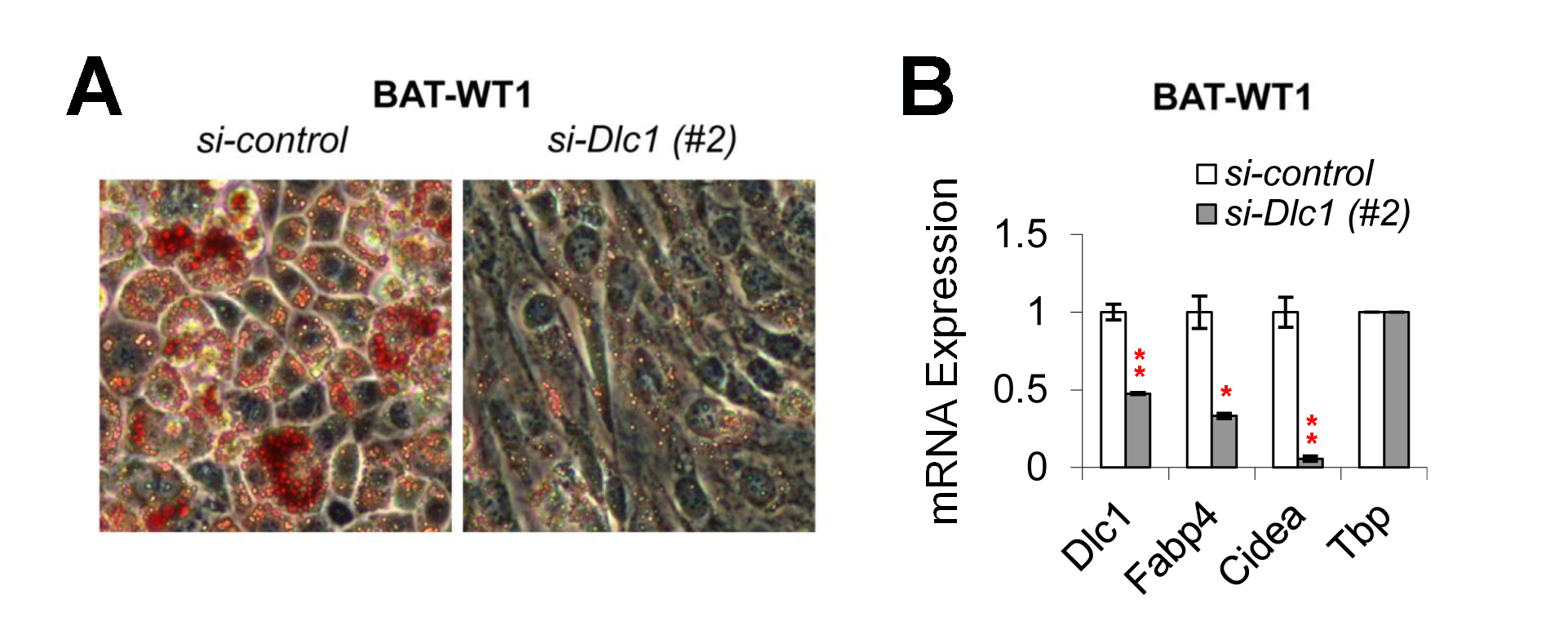

Supplement: S1 Fig — (A) Oil-Red-O staining showing reduced lipid droplet formation in Dlc1 knockdown BAT-WT1 brown adipocytes using a second Dlc1 siRNA. (B) mRNA expression of Dlc1, general adipogenic, BAT-specific genes in BAT-WT1 brown adipocytes upon Dlc1 knockdown using a second Dlc1 siRNA. Data are presented as mean ± s.e.m. n = 3 biological replicates. Two-tailed Student’s t-test was used: * P < 0.05, ** P < 0.01. (TIF) [file pone.0174761.s001.tif]

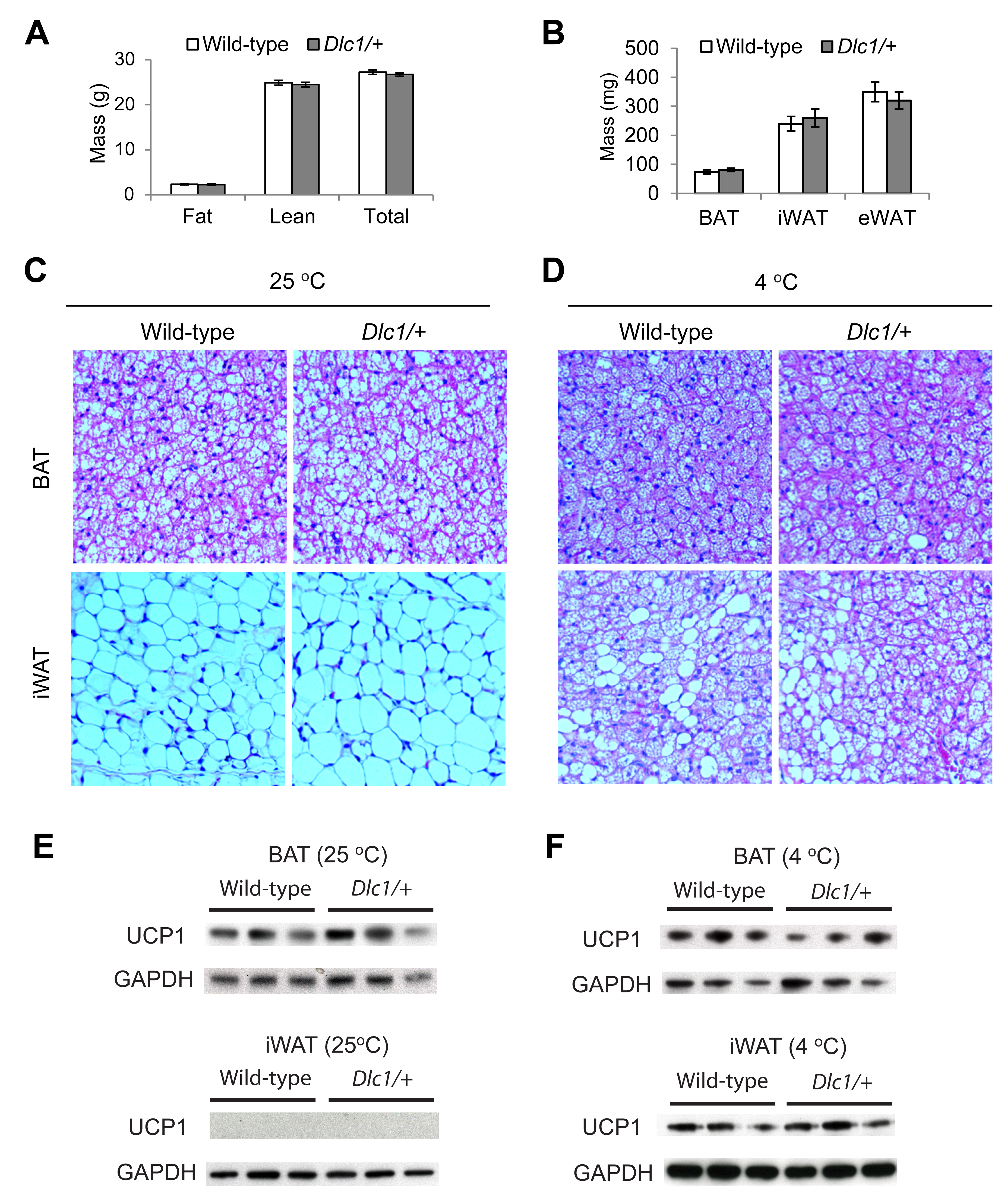

Supplement: S2 Fig — (A) Fat mass, lean mass, and total body mass of 8-week-old male Dlc1/+ and wild-type mice. n = 10. (B) Mass of BAT, inguinal WAT (iWAT), and epididymal WAT (eWAT). (C-D) H&E staining of BAT and iWAT of mice kept at 25°C (C) or 1 week at 4°C (D). (E-F) Western blot showing UCP1 level in BAT and iWAT of mice kept at 25°C (E) and 1 week at 4°C (F). (TIF) [file pone.0174761.s002.tif]

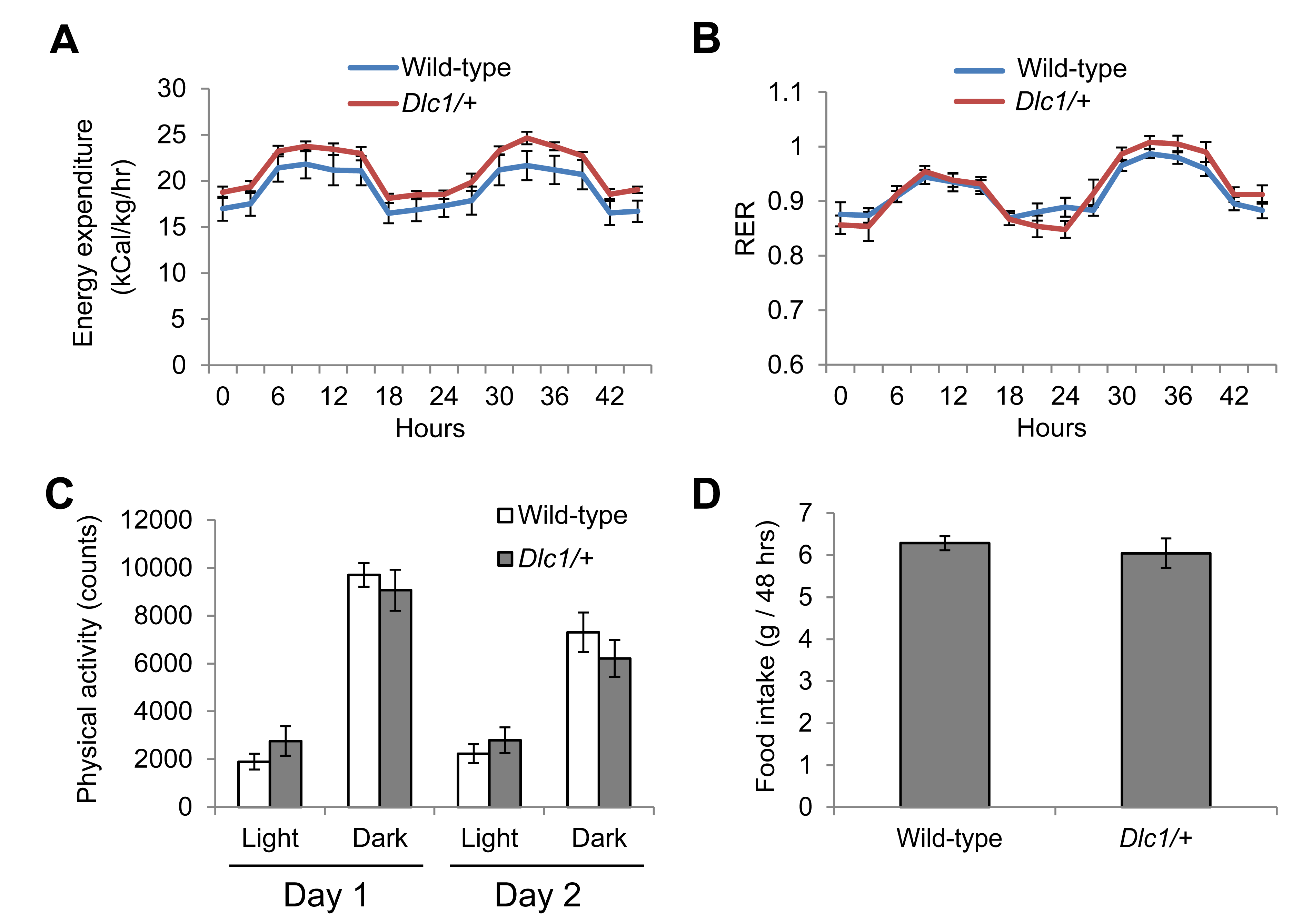

Supplement: S3 Fig — (A-D) Energy expenditure (A), respiratory exchange ratio (RER) (B), physical activity (C), and food intake (D) in wild-type and Dlc1/+ mice. n = 6. (TIF) [file pone.0174761.s003.tif]
